# Prescription Errors in a German Psychiatry Institution following a Cyber Attack: a Case Series

**DOI:** 10.1192/j.eurpsy.2026.10974

**Published:** 2026-07-31

**Authors:** J. Eberlein, K. Manaridou, O. Konstantakopoulou, G. Charalambous, C. Dokos

**Affiliations:** 1 St. Josef Psychiatric Hospital AMEOS, Oberhausen, Germany; 2 Department of Nursing, School of Health Sciences, Frederick University, Nicosia, Cyprus; 3 Department of Acute and Emergency Medicine, Klinikum Luedenscheid, Luedenscheid, Germany

## Abstract

**Introduction:**

Cyberattacks on hospitals have become increasingly common in recent years, placing a substantial strain on clinical operations. Electronic prescribing systems are particularly vulnerable, as their breakdown can interrupt routine treatment and potentially compromise patient safety. Compared with other sectors, healthcare continues to show significant deficits in cybersecurity preparedness (Kruse CS, et al. Technol Health Care 2017; 25:1–10). In Germany, several ransomware incidents have drawn attention to weaknesses in hospital IT infrastructures and their consequences for patient care (Klick J, et al. CyCon 2021; 1–10). Nevertheless, little is known about how psychiatric hospitals respond to such disruptions and what fallback strategies may ensure safe prescribing when electronic systems fail.

**Objectives:**

To evaluate the feasibility, safety, and error rates of paper-based prescribing following a cyberattack.

**Methods:**

On 7^th^ July 2025, electronic prescribing was suspended at AMEOS St. Josef Psychiatric Hospital in Oberhausen due to a cyberattack. Prescription records of 250 inpatients were retrospectively reviewed across two weekends of mandatory paper-based prescribing and the subsequent month. Prescription errors (PEs) were identified and classified using adapted EQUIP methodologies, with severity rated on a 1–4 scale and categorized into ten error types. Drug groups were classified according to the British National Formulary (BNF). A descriptive analysis was performed.

**Results:**

Two minor transcription PEs were found under BNF 10 classification. The first involving a non-steroidal anti-inflammatory drug in the first weekend and the second an analgesic drug in the following weekend. No further PEs were observed throughout the following month in contrast with previous findings (unpublished results). The transition to paper-based workflows was smooth, with staff adapting quickly and treatment continuing without delays or adverse effects.

**Conclusions:**

Paper-based prescribing provided a safe and effective fallback during cyberattack-related downtime. PEs were rare, minor, and without noticeable clinical consequences. This case series is, to our knowledge, the first to describe such a strategy within psychiatric hospitals. Routine training in manual workflows may help strengthen resilience against future cyber threats (Argaw ST, et al. BMC Med Inform Decis Mak 2019; 19:10).

**Disclosure of Interest:**

None Declared

